# Inactivation of Tnf‐α/Tnfr signaling attenuates progression of intervertebral disc degeneration in mice

**DOI:** 10.1002/jsp2.70006

**Published:** 2024-10-08

**Authors:** Chu Tao, Sixiong Lin, Yujia Shi, Weiyuan Gong, Mingjue Chen, Jianglong Li, Peijun Zhang, Qing Yao, Dongyang Qian, Zemin Ling, Guozhi Xiao

**Affiliations:** ^1^ School of Life Science and Technology Harbin Institute of Technology Harbin China; ^2^ Department of Biochemistry, School of Medicine, Shenzhen Key Laboratory of Cell Microenvironment, Guangdong Provincial Key Laboratory of Cell Microenvironment and Disease Research Southern University of Science and Technology Shenzhen China; ^3^ Department of Orthopaedics The First Affiliated Hospital of Guangzhou Medical University, Guangdong key Laboratory of Orthopaedic Technology and Implant Materials Guangzhou China; ^4^ School of Biomedical Sciences The Chinese University of Hong Kong Shatin Hong Kong; ^5^ Department of Biomedical Engineering The Hong Kong Polytechnic University Hung Hom Hong Kong; ^6^ Department of Orthopaedics, Zhujiang Hospital Southern Medical University Guangzhou China; ^7^ Shenzhen Key Laboratory of Bone Tissue Repair and Translational Research, Department of Orthopaedic Surgery The Seventh Affiliated Hospital of Sun Yat‐sen University Shenzhen China

**Keywords:** intervertebral disc degeneration, TNF receptor, tumor necrosis factor‐α

## Abstract

**Background:**

Intervertebral disc degeneration (IVDD) is a major cause of low back pain (LBP), worsened by chronic inflammatory processes associated with aging. Tumor necrosis factor alpha (Tnf‐α) and its receptors, Tnf receptor type 1 (Tnfr1) and Tnf receptor type 2 (Tnfr2), are upregulated in IVDD. However, its pathologic mechanisms remain poorly defined.

**Methods:**

To investigate the role of Tnfr in IVDD, we generated global Tnfr1/2 double knockout (KO) mice and age‐matched control C57BL/6 male mice, and analyzed intervertebral disc (IVD)‐related phenotypes of both genotypes under physiological conditions, aging, and lumbar spine instability (LSI) model through histological and immunofluorescence analyses and μCT imaging. Expression levels of key extracellular matrix (ECM) proteins in aged and LSI mice, especially markers of cell proliferation and apoptosis, were evaluated in aged (21‐month‐old) mice.

**Results:**

At 4 months, KO and control mice showed no marked differences of IVDD‐related parameters. However, at 21 months of age, the loss of Tnfr expression significantly alleviated IVDD‐like phenotypes, including a significant increase in height of the nucleus pulposus (NPs) and reductions of endplates (EPs) porosity and histopathological scores, when compared to controls. Tnfr deficiency promoted anabolic metabolism of the ECM proteins and suppressed ECM catabolism. Tnfr loss largely inhibited hypertrophic differentiation, and, in the meantime, suppressed cell apoptosis and cellular senescence in the annulus fibrosis, NP, and EP tissues without affecting cell proliferation. Similar results were observed in the LSI model, where Tnfr deficiency significantly alleviated IVDD and enhanced ECM anabolic metabolism while suppressing catabolism.

**Conclusion:**

The deletion of Tnfr mitigates age‐related and LSI‐induced IVDD, as evidenced by preserved IVD structure, and improved ECM integrity. These findings suggest a crucial role of Tnf‐α/Tnfr signaling in IVDD pathogenesis in mice. Targeting this pathway may be a novel strategy for IVDD prevention and treatment.

## Graphical abstract

The study demonstrate a critical role of the Tnf‐α/Tnfr signaling in promoting age‐ and LSI‐induced IVDD in mice. Targeting this pathway may be a novel strategy to prevent and treat IVDD development and progression.

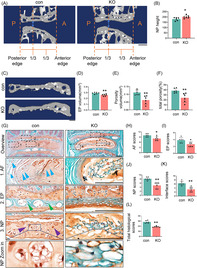

## 1 INTRODUCTION

LBP is a common and significant age‐associated disorder, which affects about 84% of the adult population.^1^, ^2^ It not only has a detrimental effect on physical function and quality of life but also causes a substantial social and economic burden.^1^ IVDD is a major cause of LBP, which is more prevalent in the elderly population.^3^, ^4^ As a typical musculoskeletal spinal condition, IVDD is influenced by multiple factors, such as aging, oxidative stress, genetic predisposition, and tissue damage from various stresses.^3^, ^4^ Incomplete understanding of the mechanism of IVDD limits current treatment approaches to physical rehabilitation surgery or pain management.^5^ Therefore, it is crucial to investigate the pathological mechanisms underlying IVDD development and progression in order to develop new treatment strategies.

IVD serves to connect the vertebrae of the spine and consists primarily of the central soft gelatinous NP, the tough outer AF, and the cartilaginous vertebral EP positioned between the NP and the vertebra.^6^, ^7^ IVD is a non‐vascular tissue that depends on the EPs as a selective permeability barrier for its metabolic processes.^8^, ^9^ The main pathological characteristics of IVDD include production of pro‐inflammatory factors, loss of ECM, increased cell senescence and death, and a decrease in the number of functional cells.^10^, ^11^ Chronic inflammation is a consequence of aging and plays an important role in age‐related diseases.^11^, ^12^, ^13^ Specifically, inflammatory cytokines TNF‐α is suspected to be an important mediator in the pathological process of IVDD.^10^, ^14^ TNF‐α accelerates IVDD via binding to TNF receptors and regulating the activity of the JNK/ERK–MAPK and NF‐kB signaling pathways in NP cells.^14^, ^15^ Administration of exogenous TNF‐α into the IVD of pigs induces early degenerative conditions in the disc, including the presence of clefts and fissures between AF lamellae, loss of NP cells, and formation of blood vessels.^16^ Meanwhile, providing TNF‐α inhibitor etanercept as a treatment for 4 weeks to patients with lumbar IVD protrusion significantly alleviated severe back pain.^17^

Although TNF‐α inhibitors and other anti‐inflammatory drugs have been utilized for treating LBP, previous studies have indicated that the early inhibition of TNF‐α in acute IVD injuries alleviated pain and may interrupt degenerative IVD processes.^18^, ^19^ In a rat model of IVD puncture injury, the timely targeting of TNF‐α led to a significant increase in paw withdrawal thresholds in the treatment groups compared to the controls, indicating a reduction in pain.^18^ Additionally, this early intervention was associated with reduced IVD degeneration, as evidenced by the preservation of tissue structure degeneration and a decrease in the expression of pro‐inflammatory cytokines in histological analysis.^18^ However, administering TNF‐α inhibitors after degeneration pain onset showed limited clinical effectiveness, highlighting potential importance of timing for anti‐TNF‐α treatment.^20^ Moreover, an infliximab TNF‐α inhibitor has a serum half‐life of 7–12 days. Although the avascular IVD might allow infliximab to stay longer within the IVD, it is unlikely that the original dose would remain active after 6 weeks.^21^ The short half‐life of TNF‐α inhibitors, combined with the complex microenvironment of degenerative disc tissue, reduces the efficacy of directly administering these drugs into the disc.^22^ Overall, their effectiveness is not considered satisfactory. The exact function of TNF‐α in IVDD is not fully understood.

In this study, we demonstrate that TNF‐α and TNFR were highly expressed in IVD from patients with severe disc degeneration, as well as in aged mice and those subjected to LSI surgery. By generating a genetic mouse model with an ablation of the genes encoding the Tnf receptors, we demonstrated that deletion of Tnfr expression notably mitigated the progression of IVDD in both age‐ and LSI‐induced mice. Specifically, Tnfr loss effectively inhibited the degradation of ECM. This study highlights the pivotal role of TNF‐α signaling in the pathogenesis of IVDD and targeting TNF/TNFR pathways could present a promising therapeutic strategy for the treatment of disc degeneration.

## 2 MATERIALS AND METHODS

### 2.1 Single‐cell RNA statistical analysis

We obtained two single‐cell RNA sequencing datasets, PRJCA014236 from the Genome Sequence Archive of the National Genomics Data Center and GSE242443, from the NCBI database. Both datasets were pre‐mapped to the GRCh38 reference genome and processed using the UMI‐tools standard pipeline to derive UMI counts for each sample. To ensure consistency with original findings, we adhered to the specific pre‐processing pipelines outlined in the respective studies for each dataset. For both datasets, only cells expressing between 200 and 7000 unique genes and with mitochondrial gene content below 10% were retained.^23^ We normalized the distinct UMI counts for each gene using the default settings in the Seurat package (v4.4.0). Subsequent dimensionality reduction and clustering analyses were conducted on the log‐normalized expression value matrix using the same package.

For the PRJCA014236 dataset, we focused our analysis on NP and AF cells. In the case of the GSE242443 dataset, our analysis targeted four distinct cell types: chondrocytes, chondroprogenitors, mesenchymal stem cells (MSCs), and proliferating MSCs.

Differentially expressed genes (DEGs) were identified using the Seurat FindMarkers function under default settings. Genes were considered differentially expressed if they had an average | log (fold change) | >0.1 and an adjusted *p*‐value <0.05, using a two‐sided Wilcoxon rank‐sum test with Bonferroni correction. We then applied the clusterProfiler package to perform KEGG pathway enrichment analysis on the identified DEGs.^24^

### 2.2 Animal studies

The *Tnfrsf1a* ^*−/−*^; *Tnfrsf1b* ^*−/−*^ mice were obtained from the Shanghai Model Organisms Center. *Tnfrsf1a* ^*−/−*^ and *Tnfrsf1b* ^*−/−*^ mice were generated in C57BL/6 genetic background mice using CRISPR/Cas9 technology. The C57BL/6 wildtype mice in the same genetic background were used as controls and 6 mice per group in this study. To conserve consistency, only male mice were used in all experiments in this study. All mice were maintained with temperatures ranging from 20 to 24°C, humidity levels between 40% and 70%, and subjected to a 12‐h light–dark cycle, with ad libitum access to water and food. We euthanized mice using carbon dioxide inhalation, and the spines were harvested for fixation in 4% paraformaldehyde (PFA). All experimental procedures on mice were approved and conducted in the specific pathogen‐free Experimental Animal Center of Southern University of Science and Technology (SUSTech‐JY202201040).

### 2.3 Genotyping

Tail‐tip tissues from mice were collected for DNA extraction and PCR (Polymerase chain reaction) according to laboratory‐established protocols.^25^, ^26^ The primers sequences for PCR were as follows: P1:5′‐AGAGCCACCAGAGACCAAGA‐3′, P2:5′‐GCCAGTGCTCTACCGTTGAT‐3′, P3: 5′‐GAAGGGGCCATGTCTATATT‐3′, P4: 5′‐AGCTACACTCACTACTCAAT‐3′, P5: 5′‐TCCTACAGCCGGATGAGCAG‐3′, P6: 5′‐CGAGCCAAACCTAAGATACC‐3′, P7: 5′‐TCAGATGTGCTGTGCTAAGT‐3′ and P8: 5′‐GACCAAGCTACACTCACTAC‐3′. Following PCR, the products were collected and subjected to electrophoresis on a 2% agarose gel at 120 V for 20 min. Images were captured post‐electrophoresis, and mice genotypes were determined based on the identification instructions provided.

### 2.4 LSI model

Following the laboratory's established protocols, 12‐week‐old C57BL/6J control or KO male mice were selected to establish either a lumbar spine instability (LSI) model or a sham procedure, with 6 mice in each group.^6^, ^27^ Briefly, mice were anesthetized with isoflurane and their dorsal fur was removed. The L3‐L4 vertebral level was located using the iliac crest line as a reference. A midline incision was made on the dorsal side, and the skin and spinous muscle tissue were sequentially dissected. After fully exposing the L3‐L5 spinous processes, the L3‐L5 spinous processes, supraspinous ligaments, and interspinous ligaments were excised to construct the LSI model. For the sham procedure, the L3‐L5 spinous processes were exposed without excision, and the incision was then closed. In addition, meloxicam was administered subcutaneously at a dose of 0.4 mg/kg for analgesia. We first used meloxicam after the mice were anesthetized with gas and continued to use it until the third postoperative day, injecting once every 24 h. At 8 weeks post‐surgery, mice were euthanized using carbon dioxide, and the spine was collected and fixed in 4% PFA.

### 2.5 Micro‐CT scanning and analysis

Following euthanasia of the mice, spines were obtained and further fixed overnight in 4% PFA. Subsequently, the μCT scanner (Skyscan1276, Bruker) was operated under the following conditions: voltage = 60 kV, current = 100 μA, and spatial scanning resolution = 10.0 μm (typ), to perform a scan of the entire lumbar spine. Image reconstruction was performed using the NRecon. Following the laboratory's established protocols, coronal images of the L3‐L4 and L4‐L5 intervertebral discs (IVDs) were used for three‐dimensional morphological analysis of the EP.^28^, ^29^ The analyzed three‐dimensional structural parameters included NP height, EP volume, porosity volume, and the percentage of porosity EP volume. The height of the NP is measured in the posterior one‐third of L4‐L5 in the sagittal plane. The EP volume was defined as the visible EP area adjacent to the vertebrae, and the analysis included 3D structural parameters such as total porosity, total tissue volume, and pore volume.

### 2.6 Histological assessment

The fixation, decalcification, dehydration, and paraffin embedding of spinal tissues were performed according to the laboratory's established protocol.^30^, ^31^ Subsequently, coronal sections of the L4‐L5 lumbar vertebrae with a thickness of 5 μm were prepared and stained with safranin O & fast green (SO&FG) staining kit (Solarbio, G1371). Following image acquisition, histological scoring was conducted in a double‐blinded manner by two independent evaluators, who were unaware of the group allocation described by Melgoza et al.^32^ Representative images were selected and presented based on the mean scores obtained.

### 2.7 Immunofluorescent analyses

Immunofluorescence (IF) staining was performed following the previously described method.^33^, ^34^ In brief, coronal sections of the L4‐L5 lumbar vertebrae with a thickness of 5 μm were subjected to deparaffinization and antigen retrieval by citrate buffer (0.1 mol·L^−1^, pH 6.0). Subsequently, sections were incubated with Triton X‐100 (Beyotime, P0096) at room temperature (RT) for 10 minutes for permeabilization, followed by blocking with Immunol Staining Blocking Buffer (Beyotime, P0102) at RT for 1 h. Next, sections were incubated overnight at 4°C with the corresponding primary antibodies as follows: TNF‐α (CST,11948 T,1:200), Tnfr1 (Abcam, ab223352,1:200), Tnfr2 (Abcam, ab109322, 1:200), Aggrecan (Abcam, ab36861, 1:200), Col2α1 (Abcam, ab34712, 1:200), Mmp13 (Abcam, ab39012, 1:200), Adamts5 (Abcam, ab41037, 1:200), Col10α1 (Abcam, ab58632, 1:200), Runx2 (CST, 12556, 1:200), Ki67 (CST, 12202S, 1:200), active caspase 8 (CST, 9429, 1:100), active caspase 3 (Sigma‐Aldrich, C8487,1:100), p53 (CST, 2524, 1:100). After washing by PBS with 0.1% Tween 20, sections were incubated with the anti‐rabbit Alexa Fluor 568 secondary antibodies at RT for 1 h. Image signals were captured using a Zeiss LSM980 confocal microscope and analyzed using Image J Java 1.8.0–172 (64‐bit). Representative images were selected based on the mean fluorescence intensity.

### 2.8 Statistical analysis

Histological scoring, IF quantification, NP height EP volume, and porosity were conducted in a blinded manner by two independent evaluators, who were unaware of the group allocation. Data analysis was conducted using GraphPad Prism 9 software. Results were presented as mean ± standard deviation (s.d.), with the sample size for each experiment indicated in the corresponding legend. To confirm the normality of the data for all variables, we conducted the Kolmogorov–Smirnov (K‐S) test. For data that were normally distributed according to the K‐S test, we utilized a two‐tailed unpaired Student's t‐test to assess the differences between the two groups. In cases where the data were not normally distributed, we employed the non‐parametric Mann–Whitney U test to determine statistical differences between the groups. Statistical significance was set at *p* < 0.05.

## 3 RESULTS

### 3.1 TNF‐α/TNFR signaling pathway is upregulated in IVD tissues in humans with severe disc degeneration and aged mice

**FIGURE 1:**
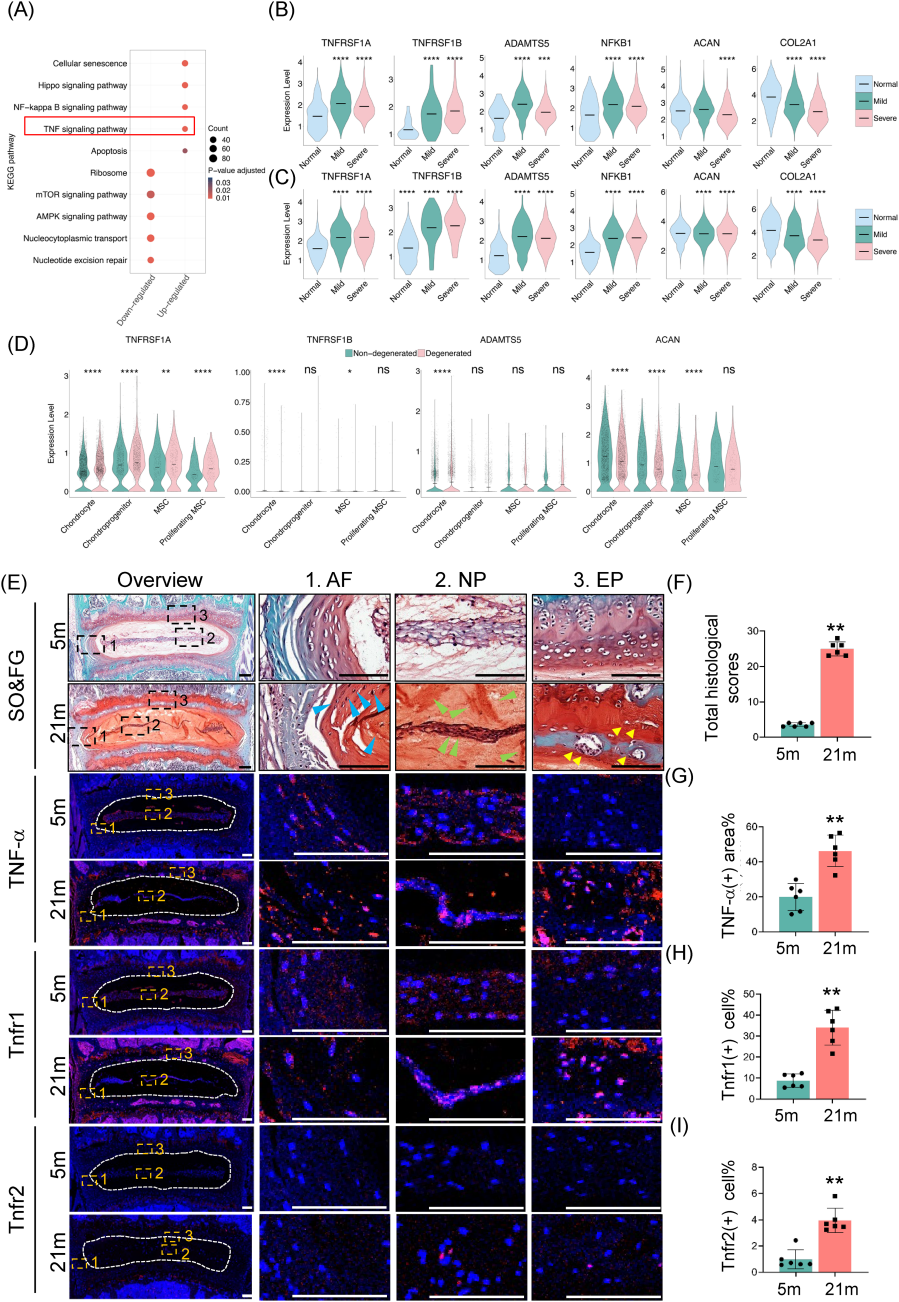
The expression of TNF signaling pathway is up‐regulated in IVD in IVDD humans and mice. (A) KEGG ontology enrichment results of up‐regulated and down‐regulated gene sets of NPCs. (B) Violin plots illustrating the expression levels of key differentially expressed genes (TNFRSF1A, TNFRSF1B, ADAMTS5, NFKB1, ACAN, and COL2A1) in NPCs between normal, mild and severe groups. (C) Violin plots illustrating the expression levels of key differentially expressed genes (TNFRSF1A, TNFRSF1B, ADAMTS5, NFKB1, ACAN, and COL2A1) in AFCs between normal, mild and severe groups. (D) Typical gene expression profiles in each cell cluster from EP (TNFRSF1A, TNFRSF1B, ADAMTS5, and ACAN), comparing non‐degenerated and degenerated groups. (E) Safranin O and Fast Green (SO&FG) staining and immunofluorescence (IF) staining of TNF‐α, Tnfr1, and Tnfr2 of lumbar IVD sections from the 5‐ and 21‐month‐old male C57BL/6 mice. Images of high magnification views of AF, NP, and EP were detailed on the right panels. White dashed lines showed the boundary between NP and AF. Scale bar, 100 μm. (F) Total histological scores of lumbar IVDs of (E). *N* = 6 mice per group. (G)–(I) Quantitative analysis of the positive areas or cells for TNF‐α (G), Tnfr1(H), and Tnfr2(I) in lumbar IVDs. *N* = 6 for each group. Results were expressed as mean ± standard deviation (s.d.). ns, no significant difference, *p <0.05, **p <0.01, ***p <0.001, ****p <0.0001.

To elucidate the molecular mechanisms driving age‐related IVDD, we conducted an analysis of single‐cell RNA sequencing data from human NP samples available in the public database (PRJCA014236).^35^ We grouped samples into normal (Pfirrmann grade I), mild (grades II and III), and severe (grades IV and V) categories of degeneration. Following the approach described by Wang et al., we identified clusters that highly expressed chondrocyte‐specific genes, such as aggrecan (ACAN) and collagen type II alpha 1 chain (COL2A1), alongside ECM genes like cartilage oligomeric matrix protein (COMP) and fibromodulin (FMOD), as NP cells, and performed KEGG pathway analysis to identify changes in cellular signaling pathways.^35^ Our results revealed a significant upregulation of the TNF signaling pathway in the severe category of disc degeneration (Figure 1A). Further exploration of DEGs highlighted substantial variations within the NPs. Notably, TNFRSF1A exhibited significantly higher expression levels compared to TNFRSF1B, with both showing increased expression in the mild and severe degeneration group (Figure 1B). Additionally, we observed an upregulation of the ECM‐degrading enzyme ADAMTS5 (a Disintegrin and metalloproteinase with thrombospondin motifs 5), along with NF‐κB1, the key component of the NF‐κB signaling pathway, while key matrix proteins, such as COL2A1 and ACAN, were downregulated in the severe category (Figure 1B). Clusters expressing the cartilage extracellular matrix genes, including COL1A1, CRTAC1, ASPN, and matrix metalloproteinase 2 (MMP2), were classified as AF cells.^35^ Similarly, in AF cells, the expression of TNFRSF1A, TNFRSF1B, ADAMTS5, and NFKB1 was notably upregulated in the severe degeneration category, while ACAN and COL2A1 were downregulated, highlighting the shift in gene expression associated with advanced disc degeneration (Figure 1C). Extending our analysis to include single‐cell RNA sequencing data of human cartilage EP from the public database (GSE242443), we categorized the main cell types into chondrocytes, chondroprogenitors, MSCs, and proliferating MSCs.^23^ DEG analysis between non‐degenerate and degenerate EP samples revealed significant expression differences. TNFRSF1A and TNFRSF1B were significantly upregulated in chondrocytes from degenerate samples, with TNFRSF1A also showing significant upregulation in chondrocyte progenitors, MSCs, and proliferating MSCs (Figure 1D). ADAMTS5 was notably upregulated in degenerate EP chondrocytes, whereas ACAN was significantly downregulated across chondrocytes, chondroprogenitors, and MSCs from degenerate samples (Figure 1D). Furthermore, we collected lumbar IVD samples from 5‐month‐old (approximately equal to 36 years old of human) and 21‐month‐old (approximately equal to 79 years old of human) male mice and performed SO&FG and IF staining.^36^ As expected, SO&FG results showed that the boundary between NP and AF was indistinct, with NP fibrosis and abnormal ossification of EP occurring in 21‐month‐old mice (Figure 1E). Histological scores of IVDs demonstrated a significant increase in 21‐month‐old mice compared to 5‐month‐old mice (Figure 1F). Immunofluorescence (IF) staining showed a significant increase in TNF‐α and TNFR1 expression in the NP, AF, and EP in older mice (Figure 1E, G, H). Notably, TNFR2 expression, though generally lower, was specifically upregulated in the IVD during degeneration (Figure 1E, I). These results suggest that Tnfr1 and Tnfr2 may play critical roles in the development and progression of IVDD.

### 3.2 Genetic deletion of Tnfr1/2 expression attenuates IVDD‐like phenotypes in aged, but not young, mice

**FIGURE 2:**
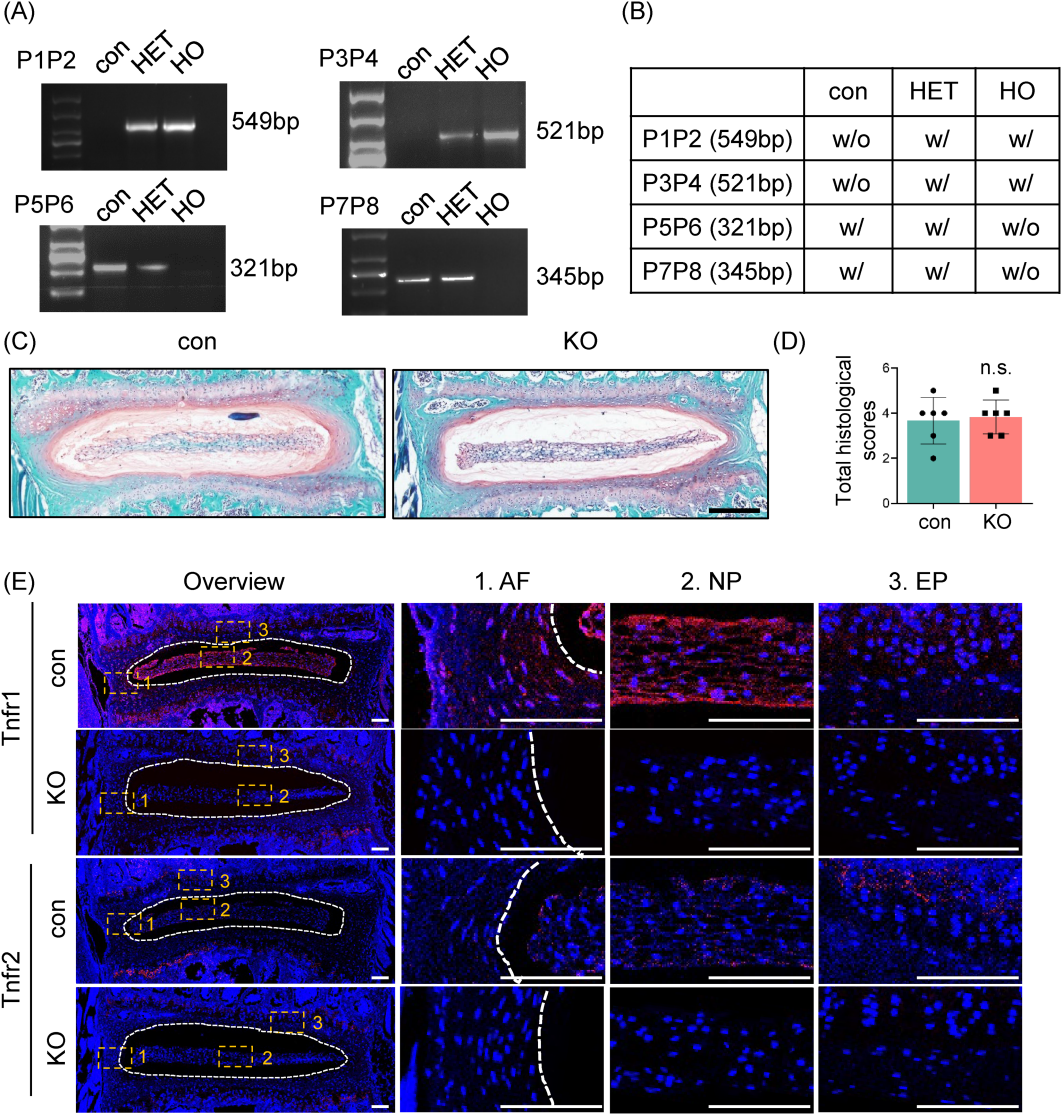
Genetic deletion of Tnfr1/2 in mice. (A) PCR genotyping using tail DNA. P1&P2: TNFR1/2 KO band, ~549 bp; wildtype (WT) showed no band; P3&P4: TNFR1/2 KO band, ~521 bp; WT showed no band; P5&P6: TNFR1/2 KO showed no band; WT band, ~321 bp; P7&P8:TNFR1/2 KO showed no band; wildtype (WT) band, ~345 bp; (B) Genotype determination table. Based on the agarose gel results (A), the mouse genotype was determined as homozygous (HO), heterozygous (HET), or control (con) according to the combinations in the table. w/o, without; w/, with. (C) SO&FG staining of lumbar IVD sections from 4‐month‐old male con and KO mice. Scale bar, 200 μm. (D) Total histological scores of lumbar IVDs of (C). *N* = 6 for each group. (E), IF staining of Tnfr1 and Tnfr2 in lumbar IVDs from 4‐month‐old male con and KO mice. Images of high magnification views of AF, NP and EP (yellow dashed boxes) were detailed on the right panels. Yellow dashed lines showed the boundary between NP and AF. Scale bar, 100 μm. Results were expressed as mean ± standard deviation (s.d.). ns, no significant difference.

**FIGURE 3:**
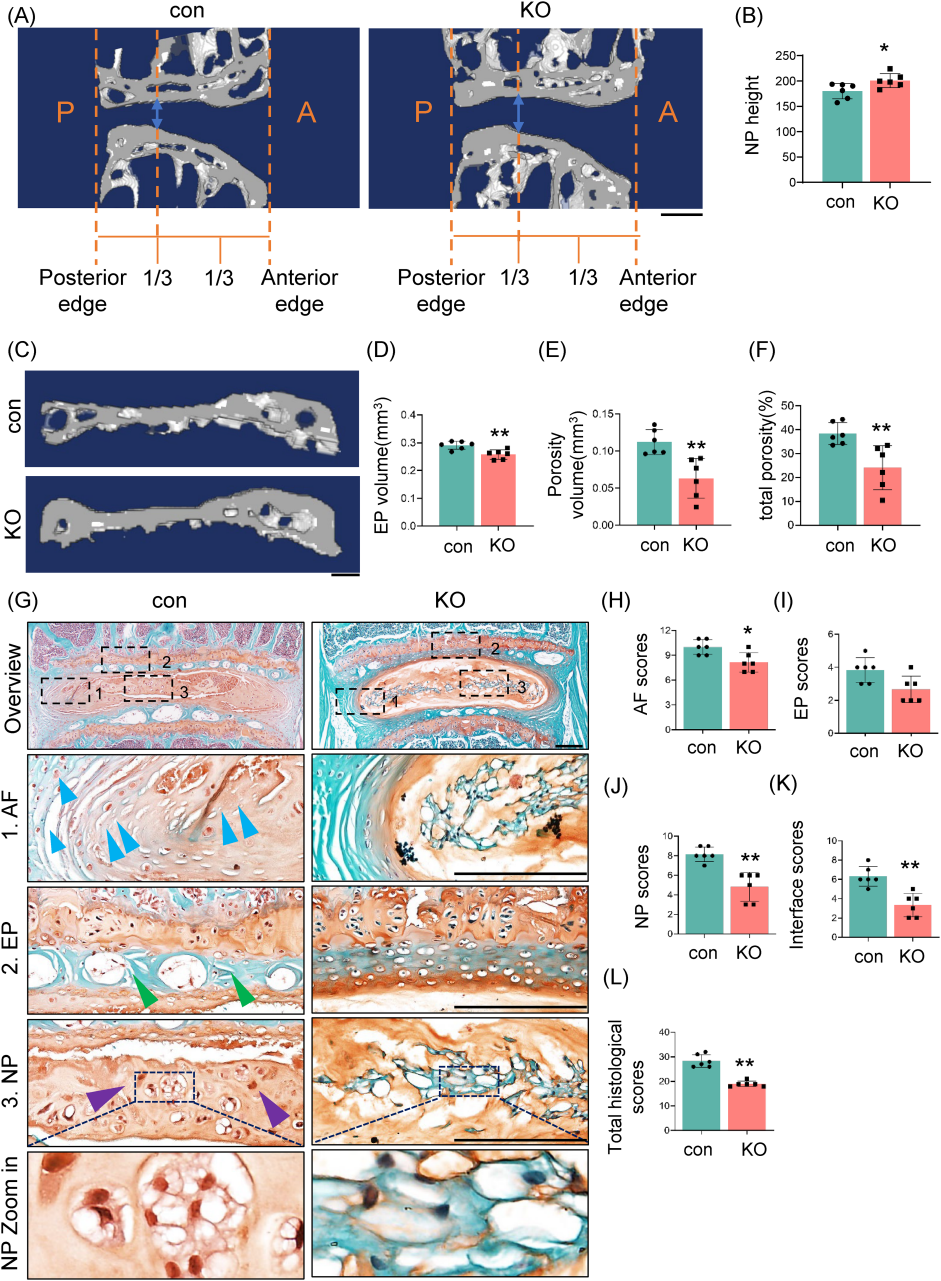
Tnfr deletion improves age‐related IVDD‐like phenotypes in lumbar IVDs in aged mice. (A) Representative sagittal μCT image of a L4‐5 lumbar IVDs from 21‐month‐old con and KO male mice. The blue arrow indicated height of NP; (P, posterior; A, anterior) scale bars, 100 μm. (B) Quantification of NP height of L4‐5 lumbar IVDs from 21‐month‐old male con and KO mice, *N* = 6 mice per group. (C) Representative three‐dimensional images of L4‐5 EPs from 21‐month‐old male con and KO mice. Scale bars, 100 μm. (D)–(F) Quantitative analysis of EP volume (D), Porosity volume (E) and the percentage of porosity (F) of L4‐5 EPs from 21‐month‐old male con and KO mice, *N* = 6 mice per group. (G), SO&FG staining of lumbar IVDs from 21‐month‐old male con and KO mice. Images of high magnification views of AF, NP, and EP (black dashed boxes) were detailed on the lower panels. Scale bar, 200 μm. (H)–(L) Evaluation of AF scores (H), EP scores (I), NP scores (J), Interface scores (K), and total histological scores (L), *N* = 6 mice per group. Results were expressed as mean ± standard deviation (s.d.). ns, no significant difference, *p <0.05, **p <0.01.

To further explore the role of TNF‐α activation in IVDD, we used a mouse line with a global deletion of Tnfr1 and Tnfr2 (referred to as KO) and wildtype mice as control group (referred to as con). Mice were in stable C57BL/6 genetic background. The PCR genotyping of the KO mice using tail DNA was determined by agarose gel electrophoresis (Figure 2A, B). The images of SO&FG staining and histological analyses of IVDs showed no significant changes in KO mice compared to control mice at 4 months of age (Figure 2C, D). As expected, IF staining of the IVDs indicated significant deletion of Tnfr1 and Tnfr2 in the AF, NP, and EP of KO mice (Figure 2E). Compared to young mice, we observed significantly increased expression of TNF signaling pathway molecules in the IVDs of aged mice (Figure 1E–I). Given these findings, we collected spinal samples from 21‐month‐old control and KO male mice. Quantitative analysis by μCT revealed a significant increase in the NP height of the L4‐5 IVD tissue in the KO group (200.77 ± 18.03 μm) compared to the control group (180.02 ± 22.75 μm) (Figure 3A, B). Additionally, the EP volume, porosity volume, and total porosity percentage of the EP were significantly decreased in KO mice compared to those in control mice (Figure 3C–F). Importantly, at 21 months of age, SO&FG staining showed milder IVDD defects in KO mice compared to control mice. Specifically, there was a more distinct boundary between the AF and NP in the KO mice (Figure 3G). The EP of the IVDs in the KO mice still exhibited densely packed chondrocyte‐like cells, while the control mice showed porous, bone‐like structures. In the NP tissue, almost all vacuolar NP cells were lost in control mice, with the hypertrophied cells visible only, while vacuolar NP cells were still present in KO mice (Figure 3G, purple arrowheads). Compared to age‐ and sex‐matched control mice, 21‐month‐old KO mice showed significantly lower histopathological scores, including AF scores (Figure 3H), EP scores (Figure 3I), NP scores (Figure 3J), interface scores (Figure 3K), and overall composite scores (Figure 3L). These results indicated that the deletion of Tnfr effectively attenuated IVDD‐like phenotypes, with significant changes observed in the AF, NP, and EP, in aged mice.

### 3.3 Tnfr deletion promotes anabolic but inhibits catabolic, metabolism of ECM in lumbar IVDs in aged mice

**FIGURE 4:**
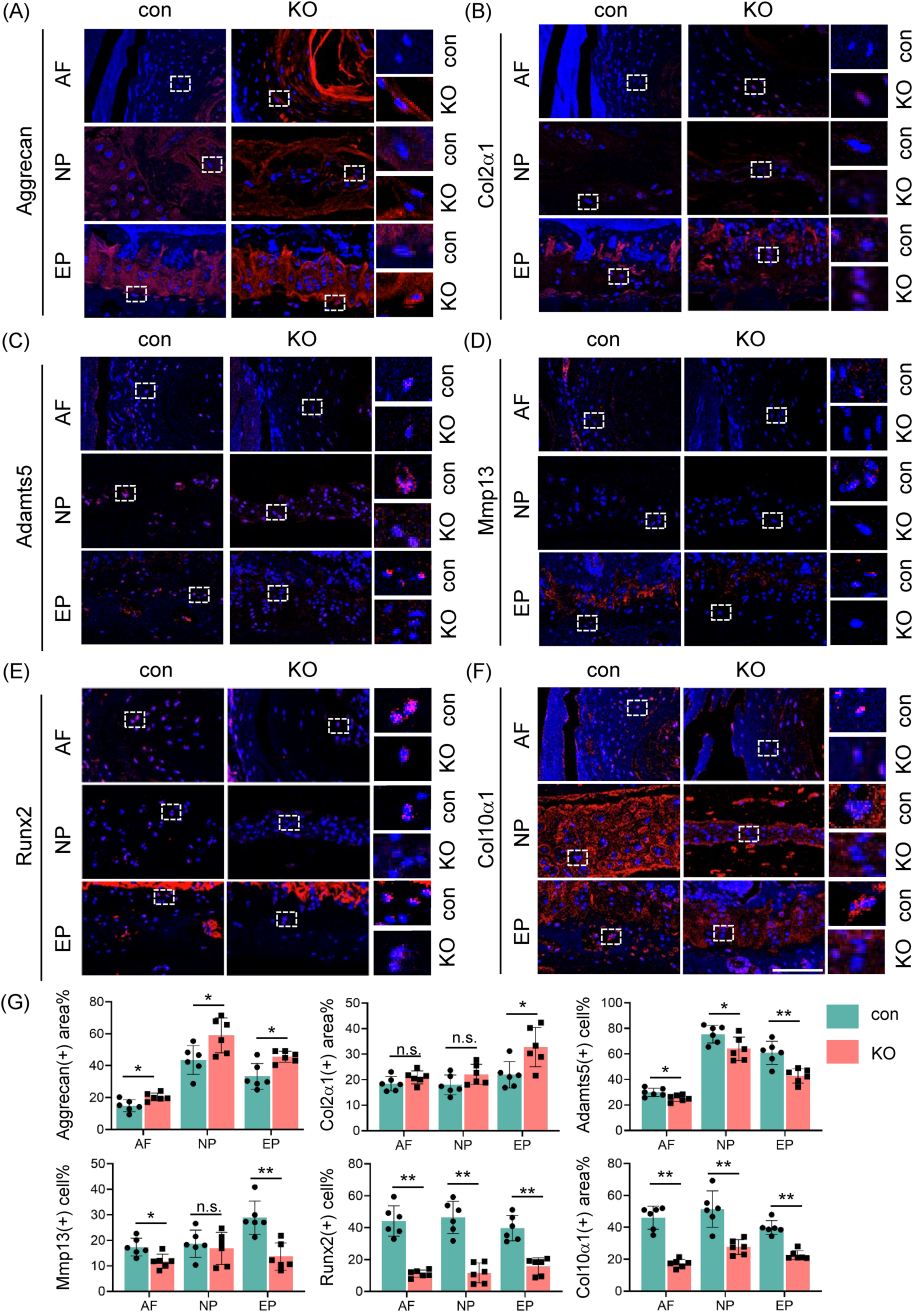
Tnfr deletion modulates ECM homeostasis in IVDs in aged mice. (A)–(F) IF staining of AF, NP, and EP of Aggrecan (A), Col2α1 (B), Adamts5 (C), Mmp13 (D), Runx2 (E), and Col10α1(F) of lumbar IVDs from 21‐month‐old male con and KO mice. Scale bars, 100 μm. (G) Quantitative analysis of the positive areas or cells for Aggrecan, Col2α1, Adamts5, Mmp13, Runx2, and Col10α in AF, NP and EP tissues. *N* = 6 mice per group. Results were expressed as mean ± standard deviation (s.d.). ns, no significant difference, *p <0.05, **p <0.01.

Results from IF staining revealed that loss of Tnfr in IVDs led to a significant upregulation of anabolic ECM proteins, including aggrecan and Col2α1 in 21‐month‐old mice (Figure 4A, B, G). Additionally, the levels of two critical ECM‐degrading enzymes, Adamts5 and matrix metallopeptidase 13 (Mmp13), were significantly downregulated in lumbar IVDs of KO mice compared to those in control mice (Figure 4C, D, G). The expression levels of proteins related to chondrocyte hypertrophy, Runt‐related transcription factor 2 (Runx2), and Collagen Type X Alpha 1 (Col10α1), which indicate the initiation and progression of EP cartilage degeneration, were also notably decreased in KO mice compared to those in control mice (Figure 4E–G). Images of the entire IVD are shown in Supplementary Figure 2. These findings suggest that Tnfr deficiency promotes ECM anabolism and inhibits its degradation, thereby decelerating the progression of IVDD.

### 3.4 Tnfr deletion inhibits cell apoptosis without affecting proliferation in IVDs in aged mice

**FIGURE 5:**
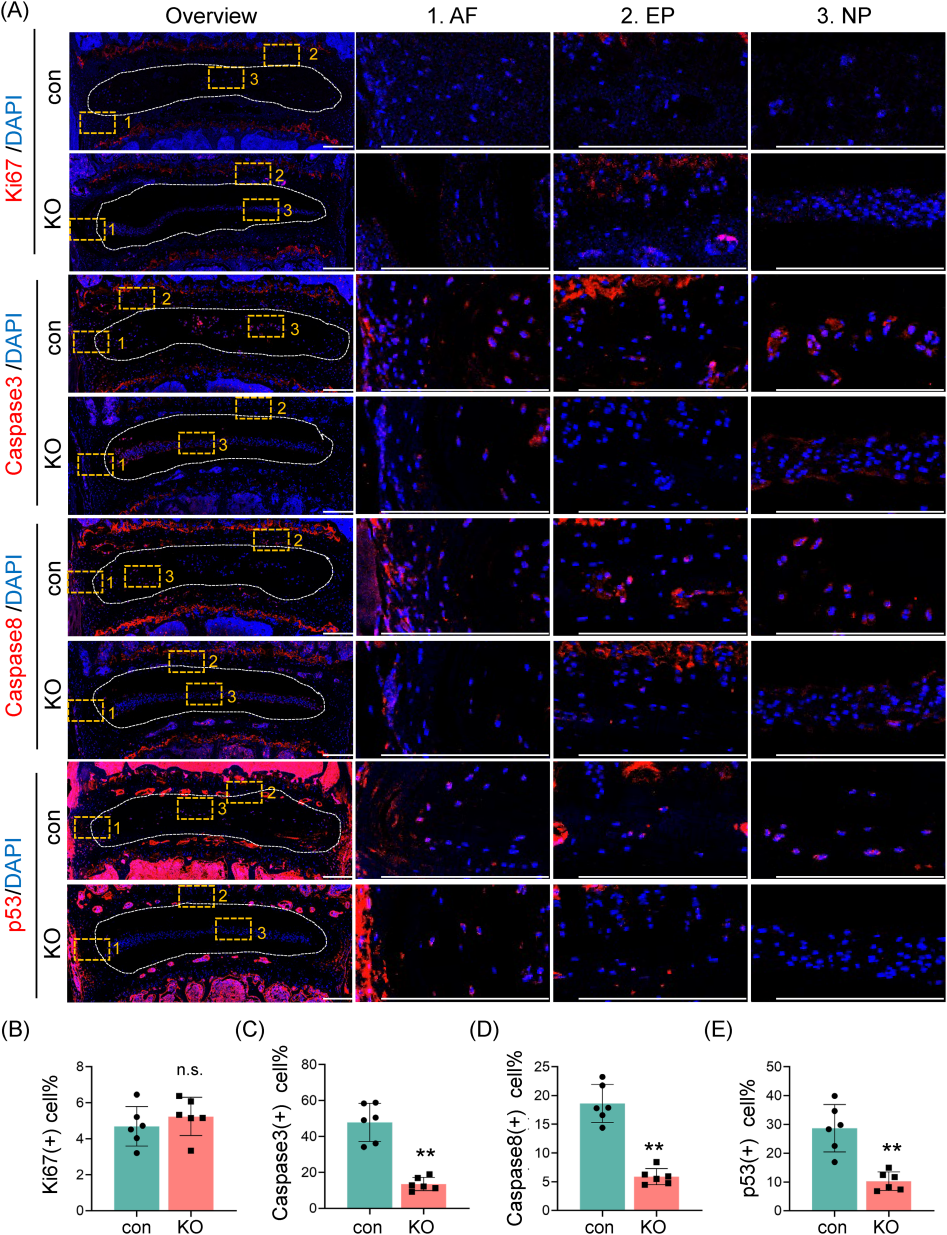
Effects of Tnfr deletion on cell proliferation and apoptosis in IVDs in aged mice. (A) IF staining of Ki‐67, Caspase3, Caspase8 and p53 of lumbar IVDs from 21‐month‐old male con and KO mice. Images of high magnification views of AF, NP, and EP (yellow dashed boxes) were detailed on the right panels. Scale bar, 200 μm. (B)–D) Quantitative analysis of the positive cells for Ki‐67 (B), Caspase3 (C), Caspase8 (D) and p53 (E) in lumbar IVDs. *N* = 6 mice per group. Results were expressed as mean ± standard deviation (s.d.). ns, no significant difference, **p <0.01.

The IF staining analyses were performed to determine the expression of the proliferation marker Ki67 and proapoptotic markers caspase‐3 and caspase‐8. Results showed that the expression of Ki67 was expressed at relatively low levels and showed no significant differences in lumbar IVDs between the 21‐month‐old control and KO mice (Figure 5A, B). However, the expression levels of caspase‐3 and caspase‐8 showed a significant decrease in the AF, NP, and EP in KO mice compared to those in control group (Figure 5A, C, D). Furthermore, we observed that the expression of p53, a cellular senescence marker, was significantly decreased in IVDs of KO mice when compared with that in control mice (Figure 5A, E). Collectively, these findings demonstrated that the loss of Tnfr reduces cell apoptosis and cellular senescence without affecting proliferation in IVD tissues in aged mice.

### 3.5 TNF signaling pathway is upregulated in LSI‐induced IVDD in mice

Spinal instability (SI), regarded as a key contributor to IVDD, often occurs after spinal fusion surgery. To investigate whether TNF signaling pathway is altered in LSI‐induced IVDD in mice, we next subjected mice to sham or LSI surgery. The results of SO&FG staining and histological scoring confirmed the successful induction of IVDD in the LSI surgery group as expected (Supplementary Figure 3A–F). IF analysis revealed a significant upregulation of the TNF signaling pathway in lumbar IVDs of LSI mice, including TNF‐α, Tnfr1, and Tnfr2, contrasted with the sham group (Supplementary Figure 3G–J). These results suggest a significant association between TNF signaling pathway activation and LSI‐induced IVDD.

**FIGURE 6:**
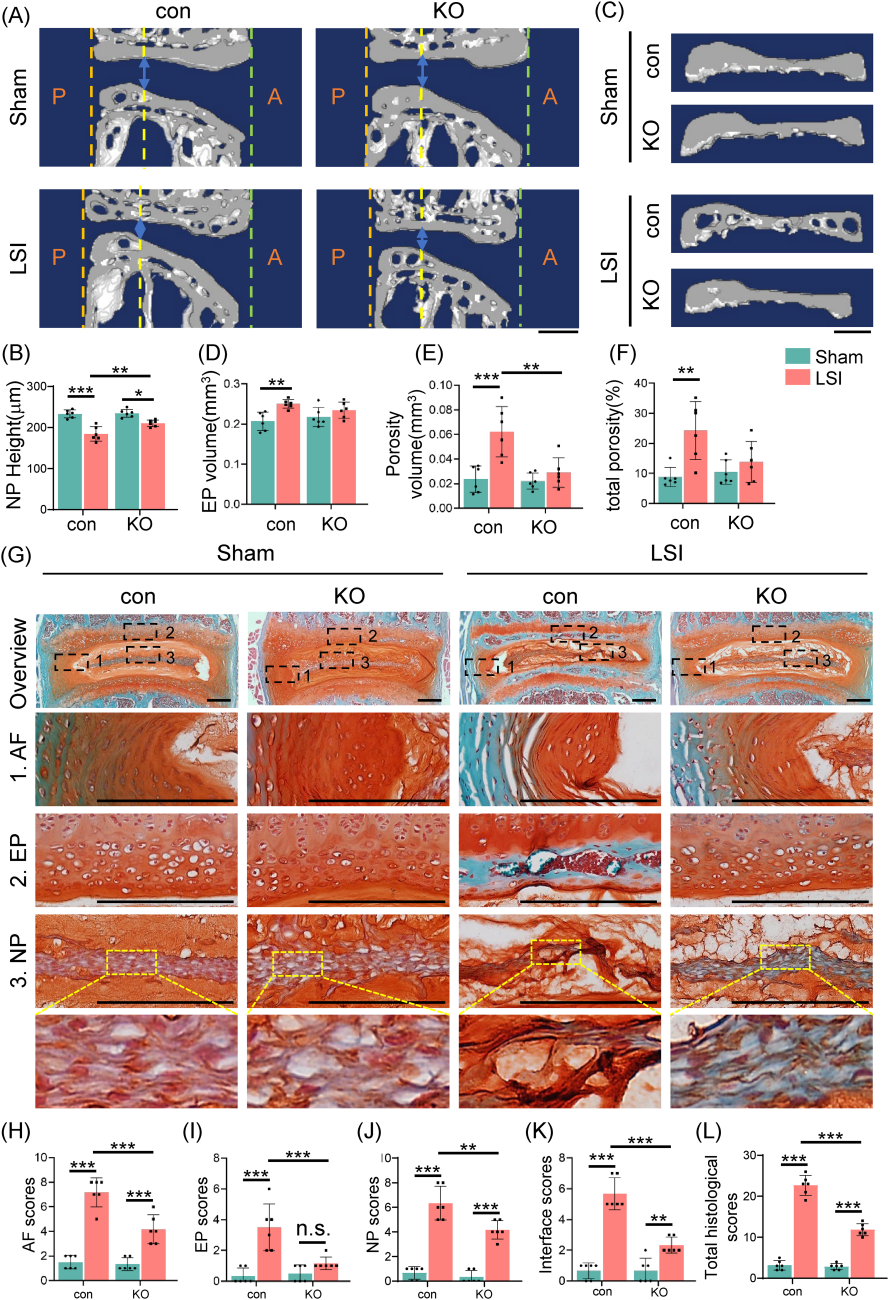
Tnfr deletion mitigates LSI‐induced IVDD‐like phenotypes in mice. (A) Representative sagittal μCT image of L4‐5 lumbar IVDs from control and KO male mice post 8 weeks of LSI or sham surgery at 3 months of age. The blue arrow indicated the height of NP (P, posterior; A, anterior), scale bars, 100 μm. (B) Quantification of NP height of L4‐5 lumbar IVDs from (A), *N* = 6 mice per group. (C) Representative 3D images of L4‐5 EPs from (A). Scale bars, 100 μm. (D)–(F) Quantitative analysis of EP volume (D), Porosity volume (E) and the percentage of porosity (F) of L4‐5 EPs from (C), *N* = 6 mice per group. (G) SO&FG staining of lumbar IVDs from control and KO male mice post 8 weeks of LSI or sham surgery at 3 months of age. Images of high magnification views of AF, EP and NP (black dashed boxes) were detailed on the lower panels. Scale bar, 200 μm. (H)–(L) Evaluation of AF scores (H), EP scores (I), NP scores (J), Interface scores (K), and total histological scores (L), *N* = 6 mice per group. Results were expressed as mean ± standard deviation (s.d.). ns, no significant difference, *p <0.05, **p <0.01, ***p < 0.001.

To further investigate the role of TNF signaling in the regulation of mechanical instability‐related IVDD, 3‐month‐old control, and KO male mice were subjected to sham or LSI surgery. Eight weeks post‐surgery, lumbar IVDs were harvested for analysis. Quantitative analysis by μCT revealed that LSI‐induced IVDD‐like phenotypes in both control and KO mice, characterized by a reduction in NP height, an increase in EP volume and porosity, and elevated histological scores of the L4‐5 IVD tissue (Figure 6A–L). Notably, deletion of Tnfr partially ameliorated these phenotypes, as evidenced by improved NP height, attenuated EP volume, and porosity, and reduced the histological scores, including NP scores, AF scores, EP scores, Interface scores, and total histological scores compared to LSI‐treated control mice (Figure 6A–L).

**FIGURE 7:**
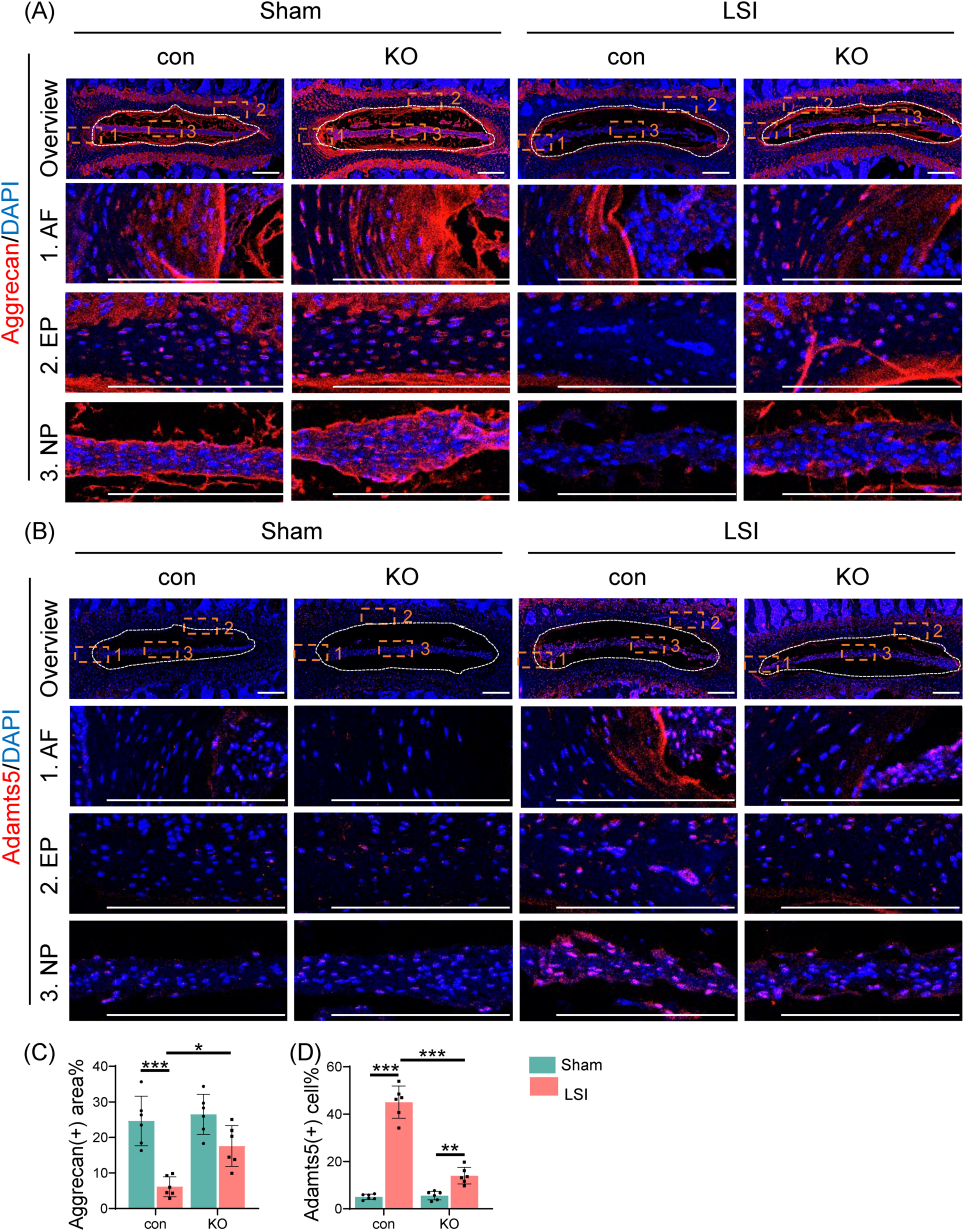
Tnfr deletion enhances ECM homeostasis in LSI‐induced IVD in mice. (A) IF staining of Aggrecan in lumbar IVDs from control and KO male mice post 8 weeks of LSI or sham surgery at 3 months of age. Images of high magnification views of AF,EP and NP (yellow dashed boxes) were detailed on the below panels. (B) IF staining of Adamts5 in lumbar IVDs from control and KO male mice post 8 weeks of LSI or sham surgery at 3 months of age. Images of high magnification views of AF,EP and NP (yellow dashed boxes) were detailed on the below panels. (C), Quantification of aggrecan‐positive areas in lumbar IVDs. *N* = 6 mice per group. (D) Quantification of Adamts5‐positive cells in lumbar. *N* = 6 mice per group. Results were expressed as mean ± standard deviation (s.d.). *p <0.05, **p <0.01, ***p <0.001.

Furthermore, results from IF staining indicated that loss of Tnfr led to upregulation of the anabolic ECM protein aggrecan and downregulation of catabolic ECM protein Adamts5 in lumbar IVDs of LSI‐treated KO mice (Figure 7A–D).

## 4 DISCUSSION

IVDD is a musculoskeletal disease associated with aging, mechanical loading, and immune inflammatory response, characterized by a reduction in the elasticity of IVDs.^37^, ^38^, ^39^ The AF surrounding the IVD becomes susceptible to cracks and tears, while the central NP starts to lose water and shrink, causing a loss of disc height.^40^, ^41^ IVDD is one of the major risk factors for LBP, but the exact mechanism is still unclear.^41^ In this study, we provide compelling evidence that inflammation may be a key factor leading to age‐ and LSI‐induced IVDD. Genetic deletion of TNF receptors significantly protects against IVDD in mice. Reanalyzing existing databases, we found that patients with severe IVDD exhibited significantly increased expression levels of TNF‐α and its receptors compared to healthy individuals and those with mild IVDD. Additionally, we found that TNF‐α and its receptors were highly expressed in aged and LSI mice compared to control mice. Our study demonstrates that IVDs of KO mice at 4 months of age exhibit a normal histological appearance similar to control mice. Interestingly, loss of Tnfr expression significantly reduced the histological scores in aged mice, increased the height of lumbar IVDs, enhanced preservation of NP cells, reduced AF ruptures and EP mineralization and decreased EP porosity in aged mice compared to age‐matched control mice. At the molecular level, compared with control mice, the absence of Tnfr increased the expression of aggrecan and Col2α1 in aged mice, decreased expression of Mmp13, Adamts5, Runx2, and Col10α1, and significantly reduced caspase‐3, caspase‐8, and p53 expression, thereby contributing to the mitigation of IVDD in aged mice. Additionally, through the LSI model, we observed that mechanical instability may contribute to the development of IVDD by upregulating inflammatory signaling pathways. Similar to age‐related IVDD, the LSI model demonstrated that Tnfr deficiency significantly ameliorated the IVDD phenotypes induced by spinal instability. These results indicate that TNF‐α/TNFR signaling plays a critical role in the pathogenesis of both age‐ and mechanically‐induced IVDD.

Mechanically‐induced IVDD is initiated by acute or chronic mechanical stress, whereas age‐induced IVDD is driven by intrinsic aging processes, including cellular senescence and telomere shortening.^32^, ^42^ Although both mechanically‐ and age‐induced IVDD involve inflammation, mechanically‐induced IVDD is characterized by acute inflammatory responses to mechanical damage, while age‐induced IVDD involves chronic and low‐grade inflammation.^32^, ^42^ Research indicates that during the aging process, IVD cells can produce TNF‐α, although it is mainly synthesized by immune cells, such as monocytes and macrophages.^28^, ^43^, ^44^, ^45^, ^46^ Previous studies have mainly focused on inhibiting TNF‐α effects through neutralizing antibodies like infliximab,^18^, ^47^ adalimumab^48^ and etanercept.^49^ Additionally, targeting downstream pathways of TNF‐α is another attractive strategy. Treatment with an IKKβ inhibitor in rats significantly alleviated the IVDD phenotypes induced by repeated puncture.^50^ However, previous studies have not investigated the direct effect of TNF‐α on aged‐related IVDD in vivo, nor have they been able to block TNF‐α completely. The immediate impact of TNF‐α on the progression of age‐related IVDD in vivo remains unclear. To explore the specific mechanisms, we reported on the histological and molecular alterations in KO mice during aging and protective role of deficient TNF‐α signaling in spontaneous IVDD. Moreover, Zeng et al. showed that lumbar instability can activate the TNF‐α signaling pathway within the IVDs.^51^ In this study, we observe that in LSI‐induced IVDD, Tnfr deficiency reduces the expression of Adamts5 while significantly increasing the expression of aggrecan, ultimately leading to decreased ECM degradation. This finding suggests that Tnf‐α/Tnfr signaling is a key mediator of ECM homeostasis in response to abnormal spinal mechanical stress.

Analysis of single‐cell sequencing data from degenerated NP, AF, and EP tissues suggests a significant increase in TNF‐α during all of IVD degeneration. This aligns with previous in vitro studies where TNF‐α treatment induced apoptosis in rabbit NP cells.^52^ Notably, results from this study also indicate that Tnf‐α/Tnfr signaling pathway is significantly activated in NP and AF in aged and LSI mice. Recently, the EP structure has been highlighted as a critical regulatory factor in pain and the aging process of IVDs.^53^, ^54^ EPs serve as crucial interface between IVDs and the vertebra, comprising of bone and cartilage layers that contribute to bearing mechanical stress and maintaining a selective osmotic barrier.^55^ During IVDD, the EP undergoes a transformative process in which chondrocyte hypertrophy is initially observed, followed by calcification and eventual resorption by osteoclasts.^56^, ^57^ This sequence of events results in the formation of a porous bone structure, which attenuates the mechanical support and nutrient exchange of the EP, thereby accelerating the IVDD.^57^ Moreover, the structural changes within the EPs are implicated in increasing nerve density and the release of neurotrophic factors such as netrin‐1, which are known to exacerbate pain sensation.^58^ We observed increased volume and porosity of the EPs in LSI and aged control mice, while KO mice displayed significant reductions in bone volume and porosity. Additionally, KO mice showed decreased expression of chondrocyte hypertrophy and degradation, including Mmp13, Adamts5, Runx2, and Col10α1. EP degradation was notably alleviated in KO mice, which contributed to maintaining mechanostructural stability and nutritional homeostasis of the IVDs. Previous research suggested that anti‐osteoclastogenic medications could effectively attenuate LSI‐induced IVDD by inhibiting EP porosity in mice.^6^ Whether KO mice can be protected from EP degradation by inhibiting the osteoclastic activities of TNF‐α requires further investigations.

We acknowledge that there are several limitations in our study. While TNF‐α plays a pivotal role in IVDD, various pro‐inflammatory cytokines, such as IL‐1β, IL‐6, IL‐10, IL‐17, chemokines, and NLRP3, also contribute to degeneration.^10^, ^59^, ^60^ Analyzing the interplay among these cytokines and their collective impact on IVD could reveal novel therapeutic targets. Understanding the inflammatory milieu in degenerative discs will clarify the intricate cytokine network involved. Additionally, Oxidative stress is also a common feature in both age‐ and LSI‐induced of IVDD. Mechanically‐induced IVDD can be caused by oxidative stress from acute mechanical injury, while age‐induced IVDD is related to cumulative oxidative damage over time.^61^, ^62^ It remains unclear whether TNF‐α/Tnfr signaling is involved in redox homeostasis in IVDD. Our findings underscore elevated levels of TNF‐α, Tnfr1, and Tnfr2 in IVDs of mice in the LSI model. Future investigation needs to explore the global role of TNF‐α in LSI‐induced IVDD beyond ECM homeostatic regulation, including its interactions with oxidative stress and the broader cytokine network.

In conclusion, we demonstrate the therapeutic efficacy of TNF‐α inhibition in IVDD. KO mice exhibit reduced degenerative alterations in IVDs compared to control mice in aged and LSI mice. Understanding molecular impact by TNF‐α on IVDs homeostasis is crucial for developing targeted treatments for age‐related IVDD.

## AUTHOR CONTRIBUTIONS

*Study design*: GX and CT. *Study conduct and data collection*: CT, SL, WG, MC, JL, PZ, and DQ. *Data analysis*: CT, SL, YS, QY, and ZL. *Data interpretation*: GX and CT. *Drafting the manuscript*: GX and CT. CT takes the responsibility for the integrity of the data analyses.

## FUNDING INFORMATION

National Natural Science Foundation of China Grants, Grant/Award Number: 82230081, 82250710175, 82261160395, 82430078, 81991513; Shenzhen Key Basic Research and Development Program, Grant/Award Number: JCYJ20220818100617036; National Key Research and Development Program of China Grants, Grant/Award Number: 2019YFA0906004; Guangdong Provincial Science and Technology Innovation Council Grant, Grant/Award Number: 2017B030301018; Shenzhen Key Laboratory of Cell Microenvironment Grant, Grant/Award Number: ZDSYS20140509142721429.

## CONFLICT OF INTEREST STATEMENT

The authors declare that they have no competing financial interests.

## Supporting information

Supplementary Figures:

## Data Availability

All data are available in the main text.

## References

[1] Balagué F , Mannion AF , Pellisé F , Cedraschi C . Non‐specific low back pain. Lancet. 2012;379(9814):482‐491. 21982256 10.1016/S0140-6736(11)60610-7

[2] Zhou T , Salman D , McGregor AH . Recent clinical practice guidelines for the management of low back pain: a global comparison. BMC Musculoskelet Disord. 2024;25(1):344. 38693474 10.1186/s12891-024-07468-0 PMC11061926

[3] Chen S , Chen M , Wu X , et al. Global, regional and national burden of low back pain 1990‐2019: a systematic analysis of the global burden of disease study 2019. J Orthop Transl. 2022;32:49‐58. 10.1016/j.jot.2021.07.005 PMC8639804 34934626

[4] Wu X , Lin S , Liao R , et al. Brief research report: effects of pinch deficiency on cartilage homeostasis in adult mice. Front Cell Dev Biol. 2023;11:1116128. 36743414 10.3389/fcell.2023.1116128 PMC9892552

[5] van Uden S , Silva‐Correia J , Oliveira JM , Reis RL . Current strategies for treatment of intervertebral disc degeneration: substitution and regeneration possibilities. Biomater Res. 2017;21:22. 29085662 10.1186/s40824-017-0106-6 PMC5651638

[6] Hu H , Chen Y , Huang F , et al. Panax notoginseng saponins attenuate intervertebral disc degeneration by reducing the end plate porosity in lumbar spinal instability mice. JOR Spine. 2021;4(4):e1182. 35005448 10.1002/jsp2.1182 PMC8717113

[7] Wu X , Chen M , Lin S , et al. Loss of pinch proteins causes severe degenerative disc disease‐like lesions in mice. Aging Dis. 2023;14(5):1818‐1833. 37196110 10.14336/AD.2023.0212 PMC10529740

[8] Wang Z , Chen H , Tan Q , et al. Inhibition of aberrant Hif1α activation delays intervertebral disc degeneration in adult mice. Bone Res. 2022;10(1):2. 34983922 10.1038/s41413-021-00165-x PMC8727577

[9] Ling Z , Crane J , Hu H , et al. Parathyroid hormone treatment partially reverses endplate remodeling and attenuates low back pain in animal models of spine degeneration. Sci Transl Med. 2023;15(722):eadg8982. 37967203 10.1126/scitranslmed.adg8982 PMC13749860

[10] Li Z , Yang H , Hai Y , Cheng Y . Regulatory effect of inflammatory mediators in intervertebral disc degeneration. Mediators Inflamm. 2023;2023:6210885. 37101594 10.1155/2023/6210885 PMC10125773

[11] Xiang H , Zhao W , Jiang K , et al. Progress in regulating inflammatory biomaterials for intervertebral disc regeneration. Bioact Mater. 2024;33:506‐531. 38162512 10.1016/j.bioactmat.2023.11.021 PMC10755503

[12] Swamy G , Salo P , Duncan N , Jirik F , Matyas J . IL‐1Ra deficiency accelerates intervertebral disc degeneration in C57BL6J mice. JOR Spine. 2022;5(2):e1201. 35783913 10.1002/jsp2.1201 PMC9238285

[13] Xia Q , Zhao Y , Dong H , et al. Progress in the study of molecular mechanisms of intervertebral disc degeneration. Biomed Pharmacother. 2024;174:116593. 38626521 10.1016/j.biopha.2024.116593

[14] Meng Q , Liu K , Liu Z , et al. Digoxin protects against intervertebral disc degeneration via TNF/NF‐κB and LRP4 signaling. Front Immunol. 2023;14:1251517. 37790932 10.3389/fimmu.2023.1251517 PMC10544936

[15] Zhang J , Wang X , Liu H , et al. TNF‐α enhances apoptosis by promoting chop expression in nucleus pulposus cells: role of the MAPK and NF‐κB pathways. J Orthop Res. 2019;37(3):697‐705. 30561076 10.1002/jor.24204

[16] Kang R , Li H , Rickers K , Ringgaard S , Xie L , Bünger C . Intervertebral disc degenerative changes after intradiscal injection of TNF‐α in a porcine model. Eur Spine J. 2015;24(9):2010‐2016. 25850392 10.1007/s00586-015-3926-x

[17] Freeman BJ , Ludbrook GL , Hall S , et al. Randomized, double‐blind, placebo‐controlled, trial of transforaminal epidural etanercept for the treatment of symptomatic lumbar disc herniation. Spine. 2013;38(23):1986‐1994. 24165696 10.1097/01.brs.0000435140.61593.4c

[18] Evashwick‐Rogler TW , Lai A , Watanabe H , et al. Inhibiting tumor necrosis factor‐alpha at time of induced intervertebral disc injury limits long‐term pain and degeneration in a rat model. JOR Spine. 2018;1(2):e1014. 10.1002/jsp2.1014 PMC6022768 29963655

[19] Korhonen T , Karppinen J , Malmivaara A , et al. Efficacy of infliximab for disc herniation‐induced sciatica: one‐year follow‐up. Spine. 2004;29(19):2115‐2119. 15454701 10.1097/01.brs.0000141179.58778.6c

[20] Cohen S , Wenzell D , Hurley R , et al. A double‐blind, placebo‐controlled, dose‐response pilot study evaluating intradiscal etanercept in patients with chronic discogenic low Back pain or lumbosacral radiculopathy. Anesthesiology. 2007;107:99‐105. 17585221 10.1097/01.anes.0000267518.20363.0d

[21] Klotz U , Teml A , Schwab M . Clinical pharmacokinetics and use of infliximab. Clin Pharmacokinet. 2007;46(8):645‐660. 17655372 10.2165/00003088-200746080-00002

[22] Molinos M , Almeida CR , Caldeira J , Cunha C , Gonçalves RM , Barbosa MA . Inflammation in intervertebral disc degeneration and regeneration. J R Soc Interface. 2015;12(108):20150429. 26040602 10.1098/rsif.2015.0429 PMC4528607

[23] Kuchynsky K , Stevens P , Hite A , et al. Transcriptional profiling of human cartilage endplate cells identifies novel genes and cell clusters underlying degenerated and non‐degenerated phenotypes. Arthritis Res Ther. 2024;26(1):12. 38173036 10.1186/s13075-023-03220-6 PMC10763221

[24] Wu T , Hu E , Xu S , et al. clusterProfiler 4.0: a universal enrichment tool for interpreting omics data. Innovation. 2021;2(3):100141. 34557778 10.1016/j.xinn.2021.100141 PMC8454663

[25] Consensus development conference: Diagnosis, prophylaxis, and treatment of osteoporosis. Am J Med 1993;94(6):646‐650. 8506892 10.1016/0002-9343(93)90218-e

[26] Gao H , Zhong Y , Zhou L , et al. Kindlin‐2 inhibits TNF/NF‐κB‐caspase 8 pathway in hepatocytes to maintain liver development and function. Elife. 2023;12:e81792. 36622102 10.7554/eLife.81792 PMC9848388

[27] Liu S , Sun Y , Dong J , Bian Q . A mouse model of lumbar spine instability. JoVE. 2021;170:e61722. 10.3791/61722 33970135

[28] Phillips KL , Cullen K , Chiverton N , et al. Potential roles of cytokines and chemokines in human intervertebral disc degeneration: interleukin‐1 is a master regulator of catabolic processes. Osteoarthr Cartil. 2015;23(7):1165‐1177. 10.1016/j.joca.2015.02.017 25748081

[29] Chen M , Li F , Qu M , et al. Pip5k1γ promotes anabolism of nucleus pulposus cells and intervertebral disc homeostasis by activating CaMKII‐Ampk pathway in aged mice. Aging Cell. 2024;e14237. 38840443 10.1111/acel.14237 PMC11488325

[30] Yao Q , Gong W , Wu X , et al. Comparison of Kindlin‐2 deficiency‐stimulated osteoarthritis‐like lesions induced by Prg4(CreERT2) versus aggrecan(CreERT2) transgene in mice. J Orthop Transl. 2023;41:12‐19. 10.1016/j.jot.2023.05.005 PMC10244901 37292436

[31] Gan D , Tao C , Jin X , et al. Piezo1 activation accelerates osteoarthritis progression and the targeted therapy effect of artemisinin. J Adv Res. 2024;62:105‐117. 10.1016/j.jare.2023.09.040 PMC11331168 37758057

[32] Melgoza IP , Chenna SS , Tessier S , et al. Development of a standardized histopathology scoring system using machine learning algorithms for intervertebral disc degeneration in the mouse model‐an ORS spine section initiative. JOR Spine. 2021;4(2):e1164. 34337338 10.1002/jsp2.1164 PMC8313179

[33] Yan Q , Gao H , Yao Q , Ling K , Xiao G . Loss of phosphatidylinositol‐4‐phosphate 5‐kinase type‐1 gamma (Pip5k1c) in mesenchymal stem cells leads to osteopenia by impairing bone remodeling. J Biol Chem. 2022;298(3):101639. 35090892 10.1016/j.jbc.2022.101639 PMC8867119

[34] Lin S , Tao C , Yan Q , et al. Pip5k1c expression in osteocytes regulates bone remodeling in mice. J Orthop Transl. 2024;45:36‐47. 10.1016/j.jot.2023.10.008 PMC10943313 38495744

[35] Wang D , Li Z , Huang W , et al. Single‐cell transcriptomics reveals heterogeneity and intercellular crosstalk in human intervertebral disc degeneration. iScience. 2023;26(5):106692. 37216089 10.1016/j.isci.2023.106692 PMC10192848

[36] Wang S , Lai X , Deng Y , Song Y . Correlation between mouse age and human age in anti‐tumor research: significance and method establishment. Life Sci. 2020;242:117242. 31891723 10.1016/j.lfs.2019.117242

[37] Xu J , Shao T , Lou J , Zhang J , Xia C . Aging, cell senescence, the pathogenesis and targeted therapies of intervertebral disc degeneration. Front Pharmacol. 2023;14:1172920. 37214476 10.3389/fphar.2023.1172920 PMC10196014

[38] Yang S , Zhang F , Ma J , Ding W . Intervertebral disc ageing and degeneration: the antiapoptotic effect of oestrogen. Ageing Res Rev. 2020;57:100978. 31669486 10.1016/j.arr.2019.100978

[39] Fu F , Bao R , Yao S , et al. Aberrant spinal mechanical loading stress triggers intervertebral disc degeneration by inducing pyroptosis and nerve ingrowth. Sci Rep. 2021;11(1):772. 33437038 10.1038/s41598-020-80756-6 PMC7804398

[40] Ohnishi T , Sudo H , Tsujimoto T , Iwasaki N . Age‐related spontaneous lumbar intervertebral disc degeneration in a mouse model. J Orthop Res. 2018;36(1):224‐232. 28631843 10.1002/jor.23634

[41] Adams MA , Roughley PJ . What is intervertebral disc degeneration, and what causes it? Spine. 2006;31(18):2151‐2161. 16915105 10.1097/01.brs.0000231761.73859.2c

[42] Tamagawa S , Sakai D , Nojiri H , et al. SOD2 orchestrates redox homeostasis in intervertebral discs: a novel insight into oxidative stress‐mediated degeneration and therapeutic potential. Redox Biol. 2024;71:103091. 38412803 10.1016/j.redox.2024.103091 PMC10907854

[43] Wuertz K , Haglund L . Inflammatory mediators in intervertebral disk degeneration and discogenic pain. Global Spine J. 2013;3(3):175‐184. 24436868 10.1055/s-0033-1347299 PMC3854585

[44] Liu XG , Hou HW , Liu YL . Expression levels of IL‐17 and TNF‐α in degenerated lumbar intervertebral discs and their correlation. Exp Ther Med. 2016;11(6):2333‐2340. 27284317 10.3892/etm.2016.3250 PMC4887958

[45] Bachmeier BE , Nerlich AG , Weiler C , Paesold G , Jochum M , Boos N . Analysis of tissue distribution of TNF‐alpha, TNF‐alpha‐receptors, and the activating TNF‐alpha‐converting enzyme suggests activation of the TNF‐alpha system in the aging intervertebral disc. Ann NY Acad Sci. 2007;1096:44‐54. 17405915 10.1196/annals.1397.069

[46] Lee S , Millecamps M , Foster DZ , Stone LS . Long‐term histological analysis of innervation and macrophage infiltration in a mouse model of intervertebral disc injury‐induced low back pain. J Orthop Res. 2020;38(6):1238‐1247. 31814143 10.1002/jor.24560

[47] Likhitpanichkul M , Kim Y , Torre OM , et al. Fibrin‐genipin annulus fibrosus sealant as a delivery system for anti‐TNFα drug. Spine J. 2015;15(9):2045‐2054. 25912501 10.1016/j.spinee.2015.04.026 PMC4550557

[48] Williams NH , Jenkins A , Goulden N , et al. Subcutaneous injection of adalimumab trial compared with control (SCIATiC): a randomised controlled trial of adalimumab injection compared with placebo for patients receiving physiotherapy treatment for sciatica. Health Technol Assessment. 2017;21(60):1‐180. 10.3310/hta21600 PMC5672499 29063827

[49] Daste C , Laclau S , Boisson M , et al. Intervertebral disc therapies for non‐specific chronic low back pain: a systematic review and meta‐analysis. Ther Adv Musculoskelet Dis. 2021;13:1759720x211028001. 10.1177/1759720X211028001 PMC8287365 34349845

[50] Kobori S , Miyagi M , Orita S , et al. Inhibiting IκB kinase‐β downregulates inflammatory cytokines in injured discs and neuropeptides in dorsal root ganglia innervating injured discs in rats. Spine. 2014;39(15):1171‐1177. 24825147 10.1097/BRS.0000000000000374

[51] Zeng Q , Sun Q , Xu H , et al. Amygdalin delays cartilage endplate degeneration and improves intervertebral disc degeneration by inhibiting NF‐κB signaling pathway and inflammatory response. J Inflamm Res. 2023;16:3455‐3468. 37600226 10.2147/JIR.S415527 PMC10438437

[52] Ishibashi H , Tonomura H , Ikeda T , et al. Hepatocyte growth factor/c‐met promotes proliferation, suppresses apoptosis, and improves matrix metabolism in rabbit nucleus pulposus cells in vitro. J Orthop Res. 2016;34(4):709‐716. 26440443 10.1002/jor.23063

[53] Fields AJ , Ballatori A , Liebenberg EC , Lotz JC . Contribution of the endplates to disc degeneration. Curr Mol Biol Rep. 2018;4(4):151‐160. 30546999 10.1007/s40610-018-0105-y PMC6287619

[54] Wang Y , Videman T , Battié MC . ISSLS prize winner: lumbar vertebral endplate lesions: associations with disc degeneration and back pain history. Spine. 2012;37(17):1490‐1496. 22648031 10.1097/BRS.0b013e3182608ac4

[55] Grunhagen T , Shirazi‐Adl A , Fairbank JC , Urban JP . Intervertebral disk nutrition: a review of factors influencing concentrations of nutrients and metabolites. Orthop Clin North Am. 2011;42(4):465‐477. vii. 21944584 10.1016/j.ocl.2011.07.010

[56] Rutges JP , Duit RA , Kummer JA , et al. Hypertrophic differentiation and calcification during intervertebral disc degeneration. Osteoarthr Cartil. 2010;18(11):1487‐1495. 10.1016/j.joca.2010.08.006 20723612

[57] Bian Q , Jain A , Xu X , et al. Excessive activation of TGFβ by spinal instability causes vertebral endplate sclerosis. Sci Rep. 2016;6:27093. 27256073 10.1038/srep27093 PMC4891769

[58] Ni S , Ling Z , Wang X , et al. Sensory innervation in porous endplates by Netrin‐1 from osteoclasts mediates PGE2‐induced spinal hypersensitivity in mice. Nat Commun. 2019;10(1):5643. 31822662 10.1038/s41467-019-13476-9 PMC6904550

[59] Roberts S , Evans H , Trivedi J , Menage J . Histology and pathology of the human intervertebral disc. J Bone Joint Surg Am. 2006;88(Suppl 2):10‐14. 10.2106/JBJS.F.00019 16595436

[60] Risbud MV , Shapiro IM . Role of cytokines in intervertebral disc degeneration: pain and disc content. Nat Rev Rheumatol. 2014;10(1):44‐56. 24166242 10.1038/nrrheum.2013.160 PMC4151534

[61] Dimozi A , Mavrogonatou E , Sklirou A , Kletsas D . Oxidative stress inhibits the proliferation, induces premature senescence and promotes a catabolic phenotype in human nucleus pulposus intervertebral disc cells. Eur Cells Mater. 2015;30:89‐103. 10.22203/ecm.v030a07 26337541

[62] Koike M , Nojiri H , Ozawa Y , et al. Mechanical overloading causes mitochondrial superoxide and SOD2 imbalance in chondrocytes resulting in cartilage degeneration. Sci Rep. 2015;5(1):11722. 26108578 10.1038/srep11722 PMC4480010

