# Supplementary material for: Inactivation of Tnf‐α/Tnfr signaling attenuates progression of intervertebral disc degeneration in mice
Source: JOR Spine. 2024 Oct 8;7(4):e70006. doi: 10.1002/jsp2.70006 (PMC11461905; doi:10.1002/jsp2.70006)
Supplement: Supplementary file 1 — Supplementary Figures: [file JSP2-7-e70006-s001.pdf]

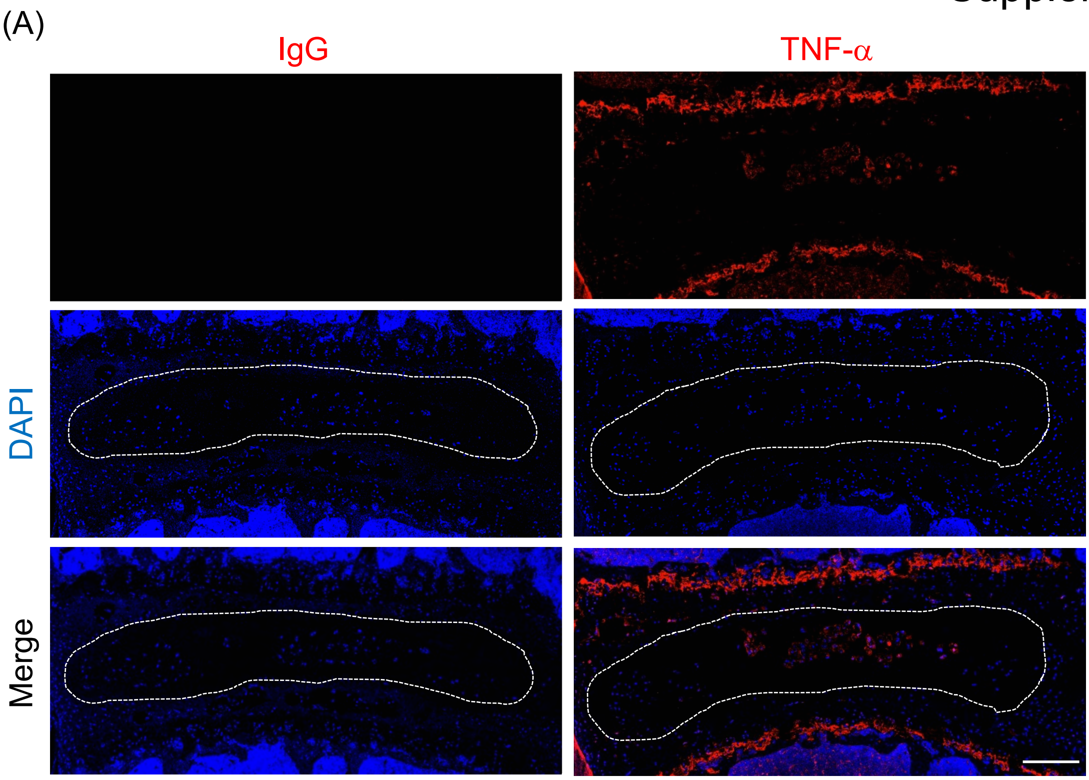

Supplementary Figure 1. Immunofluorescence (IF) staining of IgG. IF staining of IgG and TNF-α of lumbar IVD sections from the 21-month-old male C57BL/6 mice. scale bars, 200 μm.

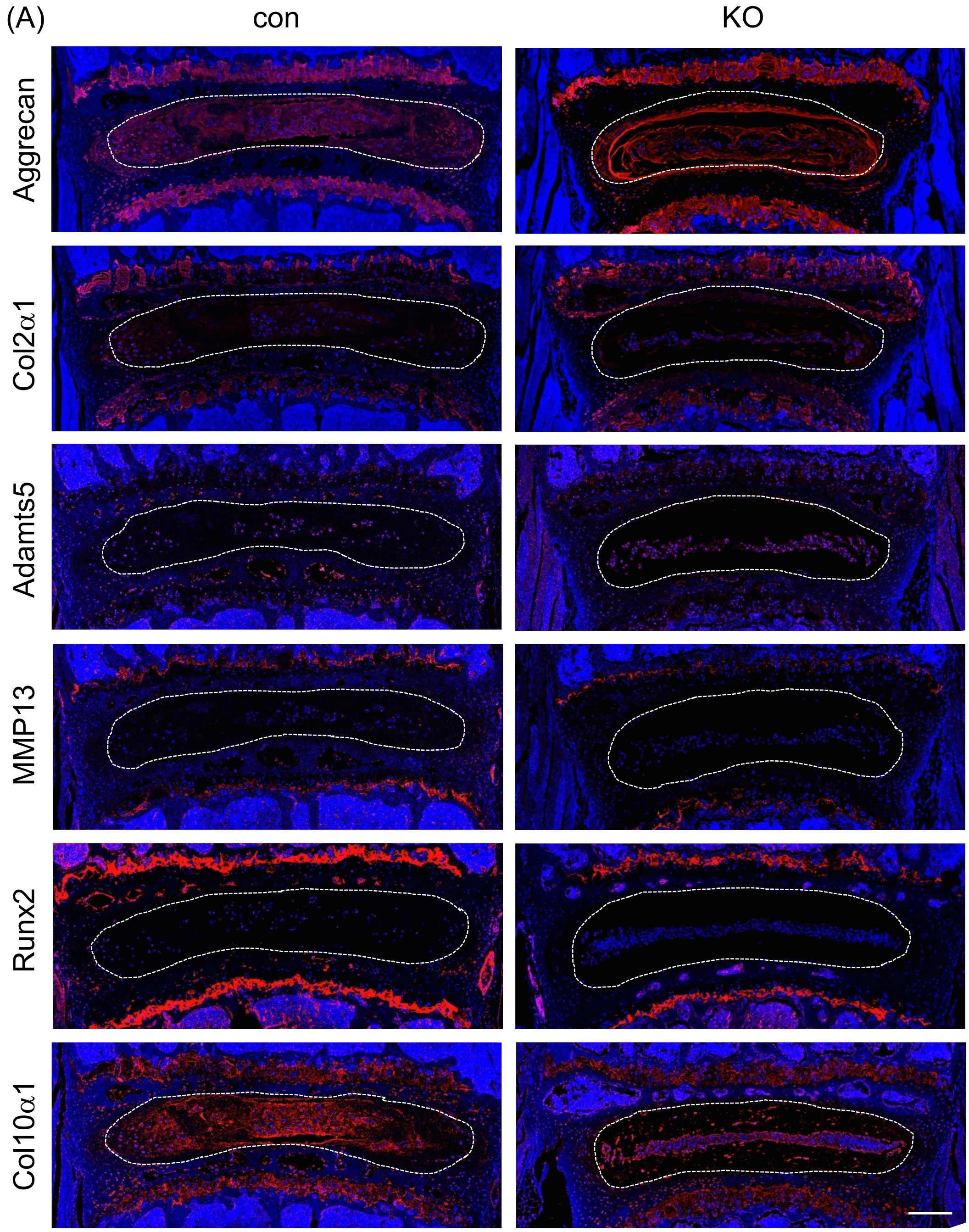

Supplementary Figure 2 Tnfr deletion modulates ECM homeostasis in IVDs in aged mice. (A) IF staining of entire IVD of Aggrecan, Col2α1, Adamts5, Mmp13, Runx2, and Col10α1 of lumbar IVDs from 21-month-old male con and KO mice. scale bars, 200 μm.

Supplementary Figure 3

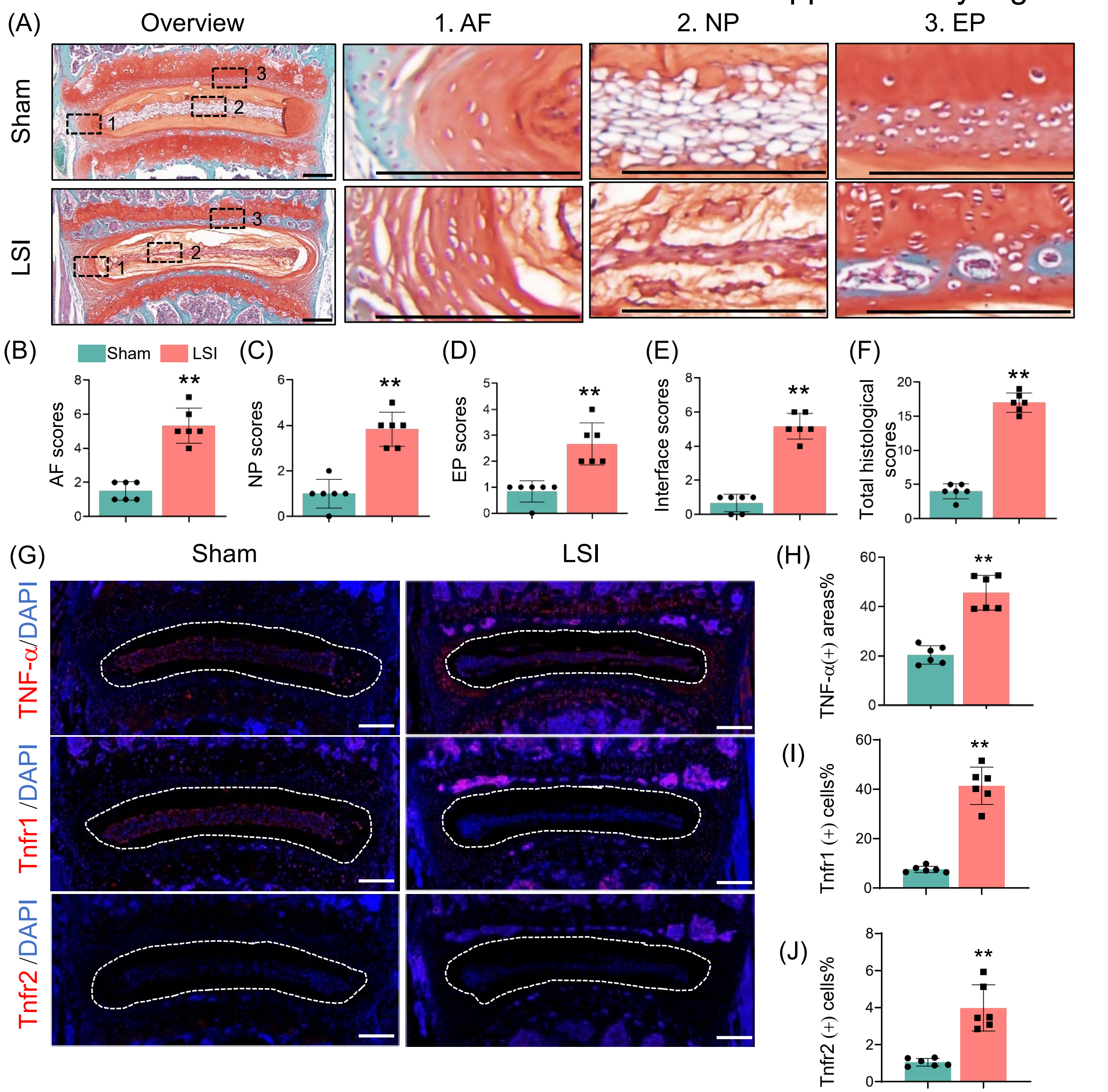

Supplementary Figure 3 TNF signaling pathway is upregulated in IVDs in the LSI model. (A) SO&FG staining of lumbar IVD sections from mice at 8 weeks after LSI surgery or sham operation. Images of high magnification views of AF, EP and NP (black dashed boxes) were detailed on the right panels. Scale bar, 200 μm. (B-F) Evaluation of AF scores (B), NP scores (C), EP scores (D), Interface scores (E), and total histological scores (F) of (A). *N* = 6 mice per group. (G) IF staining of Tnf-α, Tnfr1 and Tnfr2 of lumbar IVDs from sham and LSI group mice. Scale bar, 200 μm. (H-J) Quantitative analysis of the positive areas or cells for Tnf-α (H) , Tnfr1 (I), and Tnfr2 (J) in lumbar IVDs. *N* = 6 for each group. Results were expressed as mean ± standard deviation (s.d.). $^{**}P < 0.01$.
